# Advancements in Solid–Liquid Nanogenerators: A Comprehensive Review and Future Prospects

**DOI:** 10.3390/molecules29235716

**Published:** 2024-12-03

**Authors:** Kejie Dai, Yan Wang, Baozeng Li, Pengfei Li, Xueqing Wang, Lingxiao Gao

**Affiliations:** 1 College of Electric and Mechanical Engineering, Pingdingshan University, Pingdingshan 467000, China; daikejie@139.com (K.D.); wangyan_nwpu@163.com (Y.W.); lbz7411@163.com (B.L.); 2640@pdsu.edu.cn (X.W.); 2 School of Mechanical Engineering, Hebei University of Technology, Tianjin 300401, China

**Keywords:** solid–liquid nanogenerator, mechanisms, optimization strategies, diverse applications

## Abstract

In recent years, the advent of the smart era has confronted a novel “energy crisis”—the challenge of distributed energy provision, necessitating an imperative for clean energy development. Encompassing 71% of the Earth’s surface, water stands as the predominant conduit for energy transfer on our planet, effectively harnessing a fraction thereof to fulfill global energy demands. Modern hydropower technology primarily harnesses concentrated low-entropy water energy. However, the majority of natural water energy is widely dispersed in the environment as high-entropy distributed water energy, encompassing raindrop energy, stream energy, wave energy, evaporation energy, and other small-scale forms of water energy. While these energies are readily available, their collection poses significant challenges. Consequently, researchers initiated investigations into high-entropy water energy harvesting technology based on the electrodynamic effect, triboelectric effect, water volt effect, and other related phenomena. The present paper provides a comprehensive review of high-entropy water energy harvesting technologies, encompassing their underlying mechanisms, optimization strategies, and diverse applications. The current bottlenecks of these technologies are comprehensively analyzed, and their future development direction is prospectively discussed, thereby providing valuable guidance for future research on high-entropy water energy collection technology.

## 1. Introduction

**Figure 1:**
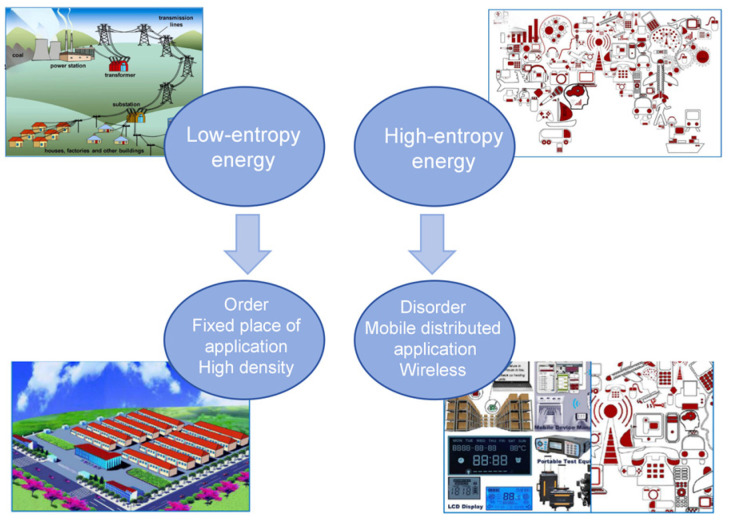
Entropy theory in power distribution. Reproduced with permission [4]. Copyright 2019, Elsevier Ltd.

With the advancement of industry as well as technological innovation, the issue of energy depletion has gained prominence. Moreover, the utilization of a substantial quantity of non-renewable resources results in the release of detrimental gases, such as sulfur dioxide, which poses an imminent threat to our environment [1]. The quest for viable alternatives to non-renewable resources has been a prominent concern among nations worldwide. Water is widely recognized as one of the most abundant resources on Earth, manifesting in diverse forms including rivers, precipitation, and atmospheric moisture [2]. Given the current global energy demand and limited reserves, it is imperative for researchers worldwide to collectively address the optimal utilization of water resources. Also, as the world enters the era of the Internet of Things (IoTs) and artificial intelligence, the traditional “orderly, centralized and high-quality” power supply mode transmitted from power plants to fixed sites such as public utilities has been difficult to meet the “random and high-entropy” energy demand of the ubiquitous Internet of Things distributed electronic devices. Therefore, random and irregular micro/nanomechanical high-entropy energy, which is widely distributed in the surrounding environment, is a promising recyclable energy source. It is imperative to develop matching collection technologies to meet the requirements of sustainable energy in the new era [3,4], as shown in Figure 1.

As early as 400 B.C., individuals commenced harnessing hydrokinetic energy. Water wheels were employed to convert the kinetic force of water into practical mechanical energy for sustenance [5]. To date, extensive research has been conducted on the extraction of mechanical or electrostatic energy from water. In 1859, Quincke made the groundbreaking discovery of streaming potential during the process of solid–liquid interactions, which involves the generation of electricity [6]. In 1867, the renowned British scientist Lord Kelvin devised the Kelvin Water Drop, an electrostatic generator specifically designed to demonstrate and elucidate the principles of voltaic theory [7]. As nanomaterials and nanotechnology continue to advance rapidly, there has been a proliferation of nanogenerators based on the synergistic interaction between two distinct materials, including solid–liquid triboelectric nanogenerators (S-L TENGs), drawing potential and waving potential based on the hydrovoltaic effect, the electrohydrodynamic effect, and the reverse electric wetting phenomenon; tribovoltaic effect-based solid–liquid nanogenerators (S-L NGs); etc. Moreover, as a next generation of energy harvesting technology, in recent years, the solid–liquid nanogenerator has been widely explored in the application field of energy harvesting devices and self-powered sensing units due to the advantages of a simple structure, low manufacture cost, high efficiency, and diverse material choices [8,9,10,11].

Triboelectric electrification (TE) is a ubiquitous event throughout daily existence, occurring across junctions with solids, liquids, and even gases [12,13]. The device effectively harnesses wasted micromechanical energy and converts it to electrical power. Evaporation accounts for a significant portion of the energy of water, which effectively absorbs thermal energy from its surroundings by transforming sensible heat into latent heat. The hydrovoltaic effect enables electricity generation through the interaction between carbon nanostructures and water in different dynamic forms, such as movement, flow, drip, and evaporation. The reverse electrowetting on dielectric (RE-WOD) technique has recently attracted interest as a potential approach for energy collection in modern research. The REWOD process can be considered as the reverse of electrowetting on dielectric (EOWD), as it effectively transforms energy from motion to electric power, exhibiting a remarkable potential for achieving exceptionally high power densities [14,15]. In addition, a novel physical phenomenon of the tribovoltaic nanogenerator (TVNG) has recently evolved, primarily due to the combination gliding of two triboelectric substances having varying electrical group energy that acts as an ongoing direct current via an exclusive mechanism [16].

**Figure 2:**
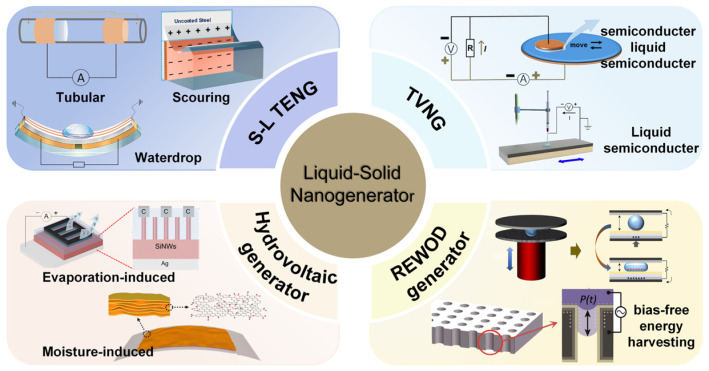
Illustration depicting the outline of this review [17,18,19,20,21,22,23,24,25].

This paper focuses on solid–liquid nanogenerators and their applications in various aspects. The conceptual structure for this assessment is shown in Figure 2. We initially examined the solid–liquid triboelectric nanogenerator, which has three modes: waterdrop, tubular, and scouring. Subsequently, we presented current advancements in enhancing the performance of S-L TENGs through techniques such as material surface modification and structure optimization. Additionally, we provided a comprehensive review of diverse mechanisms for harnessing electricity from natural evaporation and ambient moisture through an analysis of fundamental interactions occurring at various water–material interfaces. Furthermore, this review encompasses the classical electrohydrodynamic phenomena of electroosmotic flow and electrowetting, alongside the emerging advancements in reverse electrowetting and the tribovoltaic effect that have surfaced in recent years. In addition, we investigated the diverse applications of solid–liquid nano-collectors, encompassing self-powered sensors, energy harvesting systems, and various other functionalities such as self-powered anti-corrosion mechanisms, charge transfer probes for electrochemical analysis, freshwater harvesting technologies, and wearable devices. Finally, the current challenges and future research directions are summarized.

## 2. Solid–Liquid Triboelectric Nanogenerators

The solid–liquid triboelectric nanogenerator (S-L TENG) has been widely regarded as an extremely effective means of obtaining kinetic energy from liquid [26,27,28]. The operational mechanism of the S-L TENG, which relies upon the combination of both electrical phenomena with its electrical filtering operation, was extensively investigated through various previous studies [29,30,31,32,33,34,35,36]. The S-L TENG comprises three components: an electrode, a liquid triboelectric layer, and a solid triboelectric layer. The S-L TENG has three separate modes, depending on the kind of liquid triboelectric layer: waterdrop mode, tubular mode, and scouring mode.

### 2.1. Types of Solid–Liquid Triboelectric Nanogenerators

**Figure 3:**
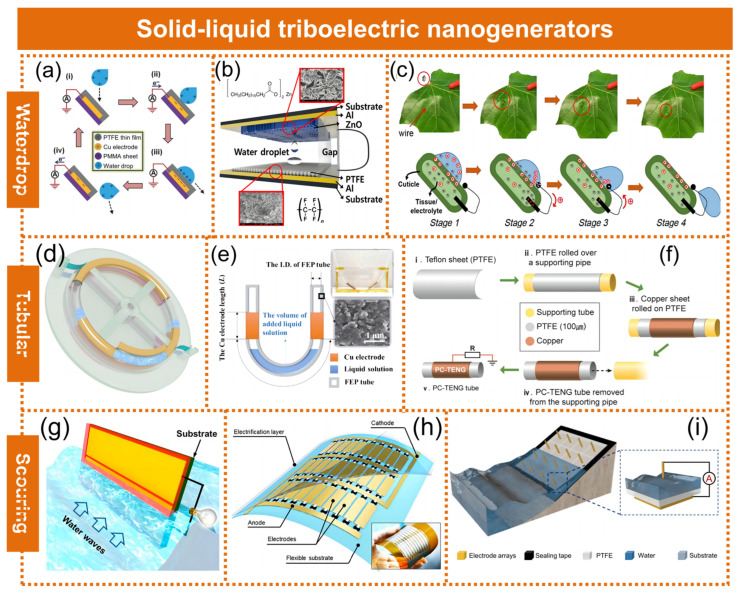
S-L TENGs with different types of triboelectric materials. (**a**) Schematic of the working mechanism of the water droplet S-L TENG with a hydrophobic PTFE layer. Reproduced with permission [37]. Copyright 2014, WILEY-VCH Verlag GmbH & Co. KGaA, Weinheim. (**b**) Schematic description of the S-L TENG showing a water droplet bouncing between two superhydrophobic surfaces of ZnO nanosheets and PTFE with Al electrodes. Reproduced with permission [38]. Copyright 2019, Elsevier Ltd. (**c**) Schematic of the working mechanism of the S-L TENG. Reproduced with permission [39]. Copyright 2020, American Chemical Society. (**d**) Schematic illustration of the ring-tube S-L TENG mounted on a rotating acrylic base with supported spokes, while two electric brushes are installed on an outer stationary acrylic base and clamped by two acrylic blocks. Reproduced with permission [40]. Copyright 2019, American Chemical Society. (**e**) Schematic illustration of the functional components of the U-tube S-L TENG. Reproduced with permission [41]. Copyright 2018, Tsinghua University Press and Springer-Verlag GmbH Germany, part of Springer Nature. (**f**) Schematic diagram of a PC S-L TENG tube based on PTFE and copper. Reproduced with permission [42]. Copyright 2022, Elsevier Ltd. (**g**) Schematic of a substrate-supported LSEG positioned in water waves. The up-and-down movement of the surrounding water body induces electricity generated between the two electrodes. Reproduced with permission [43]. Copyright 2014, American Chemical Society. (**h**) Structure of a networked integrated S-L TENG. Reproduced with permission [44]. Copyright 2018, American Chemical Society. (**i**) Schematic diagram of a bulk effect solid–liquid generator with 3D electrodes for wave energy harvesting. Reproduced with permission [45]. Copyright 2021, Elsevier Ltd.

**Waterdrop mode S-L TENG:** To harvest waterdrop energy, Wang et al. [37] prepared the droplet S-L TENG, utilizing a superhydrophobic nanostructured PTFE surface to extract energy from water droplets (Figure 3a). When a charged droplet of 30 μL contacted and separated from the PTFE surface, it generated a peak voltage of 9.3 V and a peak current of 17 μA, resulting in an instantaneous power reaching a maximum of 145 μW at 5 MΩ. Water droplets contact the surrounding environment and generate frictional charges on their surfaces. Therefore, a bouncing droplet of water has both mechanical energy and electrostatic energy. Lee et al. [38] developed a water droplet-driven solid–liquid nanogenerator that operates through triboelectric charging via contact. The S-L TENG was made out of poly tetra balls at the lower part and zinc oxide nanosheets at the upper surface, with both layers collecting energy from bouncing water droplets during contact electrification (Figure 3b). The gadget generated a brief circuit current of 1.3 μA and an open-circuit voltage of 1.4 V. Solid–liquid triboelectric nanogenerators (S-L TENGs), serving as green energy harvesters, often employ non-biodegradable plastic films. Therefore, Wu et al. [39] reported a fully biodegradable S-L TENG, which used the external cuticle as well as the inner tissue with electrical conductivity of a leaf to serve as the triboelectric substance along with the conductor, with water droplets employed as the counterpart to successfully collect energy from the droplet impact onto a plant leaf (Figure 3c).

**Tubular mode S-L TENG:** Wang et al. [40] fabricated an S-L TENG utilizing a liquid–dielectric interface for direct current generation, which demonstrated the advantages of feasible fabrication, anti-wearing durability, and low energy consumption (Figure 3d). The device, comprising an FEP tube and Cu electrodes arranged in a ring structure, was equipped with two electric brushes anchored bilaterally to convert AC output into direct current output. The fluid-state dielectrics, consisting of liquids and copper pellets, were pre-filled to induce triboelectric charges using an FEP tube. Consequently, the rotating excitations resulted in satisfactory output from the device. When the angular velocity hit 70 revolutions per minute, the maximum open-circuit voltage reached a maximum of 228 V. After applying an external load, the power output was highest at a resistance value of 120 MΩ, resulting in a top power of 3.7 mW. Pan et al. [41] constructed a U-shaped tube triboelectric nanogenerator based on the solid–liquid mode and investigated the impact of liquid characteristics on the S-L TENG’s output performance by testing 11 different liquids. The findings demonstrated that characteristics such as charge distribution, permittivity, and attraction to fluorinated ethylene propylene (FEP) were crucial factors influencing the S-L TENG’s output (Figure 3e). The device delivered the best output, with an open-circuit voltage of 81.7 V and short-circuit current of 0.26 μA for the shaking mode and open-circuit voltage of 93.0 V and short-circuit current of 0.48 μA for the horizontal shifting mode. The underutilization of the green energy present in flowing water is primarily attributed to the dearth of environmentally acceptable and sustainable technologies for harvesting such energy. Munirathinam et al. [42] developed a polytetrafluoroethylene–copper tube with a single electrode mode for harvesting flowing water energy (Figure 3f). Utilizing tap water as the primary source, the device, measuring 40 cm in length and 10 mm in diameter, achieved a peak power output of 45 µW when connected to a load resistance (RL) of 5 MΩ.

**Scouring mode S-L TENG:** Obtaining electricity from water’s surrounding motions is a promising but underutilized approach for meeting local energy needs in self-sustaining electronics. Zhu et al. [43] reported a solid–liquid triboelectric nanogenerator (S-L TENG) that utilized a film coating of fluorinated polymer to collect energy from various water motions (Figure 3g). The device generated an open-circuit voltage of 160 V and an optimum average output power of 0.12 mW at a relative velocity of 0.5 m s^−1^. The wave energy availability in the environment is not constant but appears randomly, making it difficult to harvest. To address this issue, Zhao et al. [44] proposed a solid–liquid triboelectric nanogenerator featuring an arrayed networking structure for efficient energy harvesting from diverse water wave interactions (Figure 3h). The device incorporated several sets of electrodes that were constructed onto a pliable manufactured material. The electrode material employed in their study was a conductive textile, while the hydrophobic coating utilized a dry-etched PTFE film. Due to its two-dimensional networked structure, the device exhibited exceptional adaptability to various wave motions on the water; it consistently generated a reliable electrical supply irrespective of pattern characteristics. The practical applications of the device in real circumstances, characterized by highly variable and unpredictable water waves, hold great promise in terms of their potential impact. Gu et al. [45] introduced an electrode array-based solid–liquid generator, which exhibited enhanced wave energy harvesting capabilities compared to conventional S-L TENG (Figure 3i). The primary factor lies in the three-dimensional electrode design implemented on the dielectric layer, which engendered a volumetric effect that established a closed-loop system encompassing the electrode array, polymer layer, and aqueous volume. The generator was capable of delivering voltage and current up to 42 V and 4 mA, respectively, while achieving a maximum instantaneous power of 18.36 mW with an optimal external resistance of 51 kΩ.

### 2.2. Methodology for Enhancing Performance of the Solid–Liquid Triboelectric Nanogenerator

Currently, the limited output performance of the solid–liquid triboelectric nanogenerator remains a pivotal constraint impeding its widespread adoption and application. To address the issue of low output efficiency in solid–liquid triboelectric nanogenerators, researchers have proposed diverse strategies to enhance their output performance. The advancements encompass enhancing the performance of the materials employed in its construction and optimizing the structure of the S-L TENG.

#### 2.2.1. Methodology for Optimizing Materials

**Figure 4:**
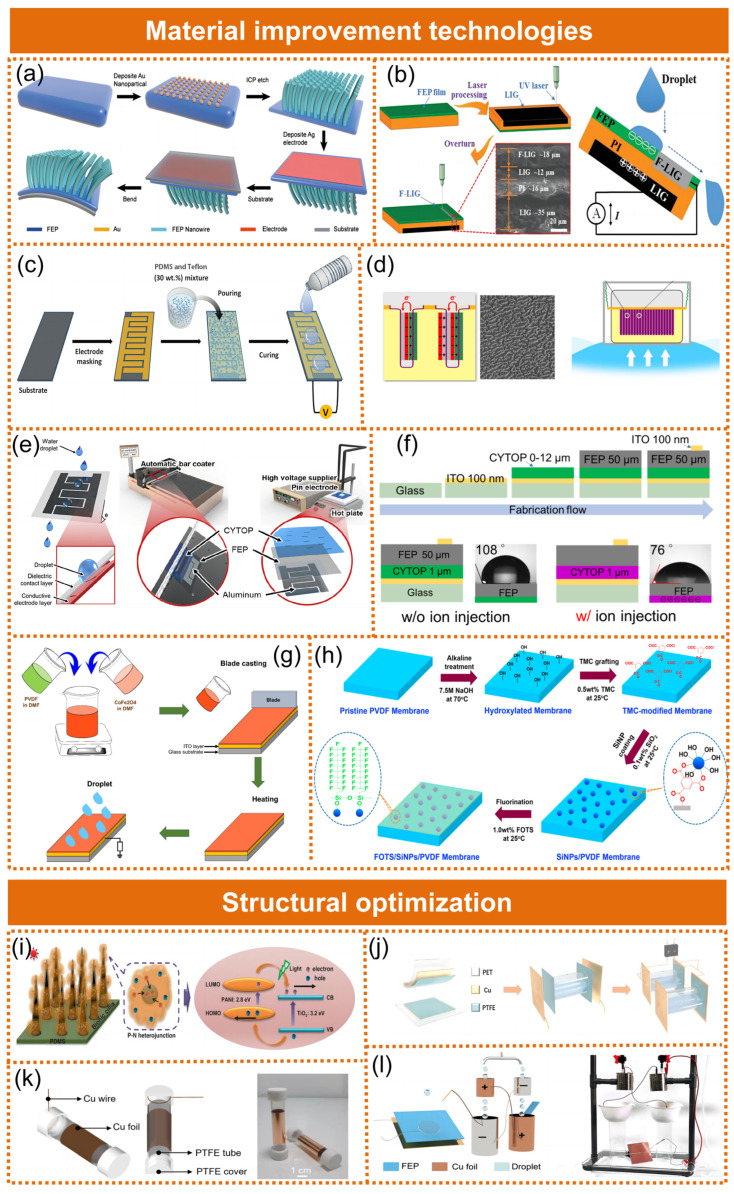
An overview of the output enhancement methods for S-L TENGs. (**a**) Fabrication steps of an S-L TENG based on nanowires. Reproduced with permission [46]. Copyright 2017, AIP Publishing. (**b**) Interface laser-induced graphene to achieve a full fabrication process as well as an operational principle for a high-performance solid–liquid triboelectric nanogenerator. Reprinted by license [47]. Copyright 2021, Wiley-VCH GmbH. (**c**) Schematic representation of the device and hybrid film (polydimethylsiloxane/polytetrafluoroethylene or PDMS/PTFE) fabrication scheme and the surface morphology of PDMS and PDMS/PTFE films with 10, 20, and 40 wt % PTFE particles. Reproduced with permission [48]. Copyright 2021, John Wiley & Sons Ltd. (**d**) The charge injection performance of the triboelectric nanogenerator is optimized by an air-driven membrane structure [49]. Copyright 2017, Elsevier Ltd. (**e**) Schematic configuration of an S-L TENG and fabrication of a CYTOP electret-based S-L TENG by using a bar coater and the corona charging method with simultaneous thermal annealing. Reprinted by license [50]. Copyright 2020, Elsevier Ltd. (**f**) Fabrication process of a CYTOP-based DEG and contact angle of the surface without and with negative ion injection [51]. Copyright 2023, IOP Publishing on behalf of the Japan Society of Applied Physics. (**g**) Schematic diagram of CFO/PVDF membrane fabrication and dielectric constants of pure PVDF and CFO/PVDF membranes at different frequencies. Reproduced with permission [58]. Copyright 2022, MDPI. (**h**) Schematic diagram of the procedure for epitaxial growth on a silica nanoparticle (SiNP)-coated and FOTS-functionalized PVDF membrane. Reprinted by license [54]. Copyright 2021, Elsevier Ltd. (**i**) Photocatalytic mechanisms of bionic cilia with a P-N junction and morphological analysis of the cilia are presented. The insets display the water contact angles of the three samples. Reproduced with permission [61]. Copyright 2020, Springer Nature. (**j**) Schematics of STVE S-L TENGs and output performance of a tube-based S-L TENG with/without end electrode. Reproduced with permission [62]. Copyright 2022, Wiley-VCH GmbH. (**k**) A block diagram of the fabrication procedure for an S-L TENG array and short-circuit current of a single S-L TENG device and integrated dual S-L TENG devices. Reproduced with permission [63]. Copyright 2020, Elsevier Ltd. (**l**) Design and performance of the K-DEG. Reproduced with permission [64]. Copyright 2024, Droplet published by Jilin University and John Wiley & Sons Australia, Ltd.

Methods for material optimization encompass physical modifications [46,47,48], electron injection [49,50,51], and chemical modifications [52,53,54]. The most commonly employed treatment method involves enhancing surface roughness through physical modification to optimize the contact area [55]. Li et al. [46] fabricated nanowires by etching (Figure 4a), which largely added to the roughness of the surface and thus magnificently increased the contacting area of the triboelectric layer and water. The output of the S-L TENG was magnificently increased. An output current of 10 µA and a voltage of about 200 V were generated by the nanowire-based S-L TENG (a contact area of 30 cm^2^). Chen et al. [47] developed the live-generating laser-induced graphene technique, which enabled the etching of extremely hydrophobic fluorine-doped carbon onto fluorinated polyethylene polypropylene (FEP)-coated polyimide (PI) (Figure 4b). The device exhibited a significantly high power conversion efficiency, resulting in the generation of a remarkable peak power density of 47.5 W m^−2^ upon impact with a water droplet measuring 105 µL and dropped from a height of 25 cm. Fuwad et al. [48] mixed polydimethylsiloxane (PDMS) along with polytetrafluoroethylene (PTFE) to generate a powerful solid–liquid film, thereby enhancing power-collecting performance (Figure 4c). The inclusion of PTFE granules in PDMS changed the material’s property of the surface, boosting roughness as well as the transfer of charge efficiency while enhancing its rigidity. During typical operation, the circuit exhibited a nominal voltage at the output of 50 V and an average power consumption of 0.675 W m^−2^ across a resistance of 2 MΩ. The most direct approach to enhancing output performances lies in the augmentation of surface charge density.

Xu et al. [49] optimized the charge injection performance of a triboelectric nanogenerator by air-driven membrane structure (Figure 4d). Nanostructures were introduced on the surface of polytetrafluoroethylene (PTFE) to enhance the contact electrification effect, and the pressure difference between the upper and lower chambers was used to drive the contact and separation of the TENG unit to achieve efficient charge transfer. High-voltage corona charging is a widely used surface charge injection method, which was initially used in solid–solid TENGs and later adapted to S-L TENGs. The injection of charge on amorphous fluoropolymers by Jang et al. [50] significantly enhanced peak power, surpassing a 1,000-fold increase (Figure 4e). Their study emphasized the advantages of using the high-voltage corona charging method, which did not affect the transmittance and flexibility of the dielectric film. In 2023, by using the ionized air charge injection method, Wang et al. successfully injected negative charges into a CYTOP layer [51]. Therefore, the surface potential of the device decreased from −0.56 V to −0.28 kV after charge injection, and the surface contact angle decreased from 108° to 76° (Figure 4f). The proposed device achieved a peak voltage and current output of 42 V and 65 μA. Vu et al. [56] fabricated a triboelectric material composed of polyvinylidene fluoride-hexafluoropropylene (PVDF-HFP) and an ionic liquid (PIL), namely, a PIL membrane. In their study, ([BMIM]^+^[TFSI]^−^) was used as the dopant ionic liquid to fabricate the PVDF-HFP/ionic liquid (PIL) membrane. The addition of the fluoride concentration caused an increase in the charge density in the triboelectric layer, which greatly enhanced the power generation capacity of S-L TENGs. Under ideal circumstances, the nanoporous PIL-TENG containing 10 wt.% of ions achieved the highest peak-to-peak voltage transfer of 16.95 V as well as amperage of 2.56 μA. Its PIL-TENG had a maximum instantaneous maximal energy density of 0.0261 W m^−2^, 212% greater than the pure PVDF-HFP TENG (P-TENG). The saturated charge density of the S-L interface was the key element to decide its performance. Tao et al. [57] found that the saturated charge density of S-L contact electrification (CE) could be further increased under the illumination of an ultraviolet (UV) light.

Several studies have corroborated that chemical modification can enhance the performance characteristics of S-L TENGs. The study conducted by Vu et al. [58] utilized magnetic copper, a mineral called CoFe_2_O_4_ (CFO), and tiny particles in conjunction with PVDF to fabricate a triboelectric hybrid barrier aimed at enhancing the output of a S-L TENG (Figure 4g). As a consequence, the output of the CFO/PVDF-5 S-L TENG increased significantly with a voltage of 17.2 V, a current of 2.27 µA, and a power density of 0.09 W m^−2^. Le et al. [54] presented a powerful S-L TENG using surface polarity adjustment and an epitaxial development on a polyvinylidene fluoride (PVDF) substrate. The PVDF material interface was functionalized on tiny particles of silica (SiNPs) by the use of chemical bonds before being seeded using a negative charge of 1H, 1H, 2H, and 2H-Perfluorooctyltriethoxysilane (FOTS) in order to form the FOTS/SiNPs/PVDF (FSiP) barrier (Figure 4h). The suggested barrier might produce fluorine-containing siloxane molecules that improve surface polarity, resulting in remarkable hydrophobicity and dielectric constant. The device’s output power displayed exceptional triboelectric performance with a current of 5.79 μA, a voltage of 28.3 V, and a maximum power density of 0.42 W m^−2^. Chen et al. [59] developed a robust single-electrode triboelectric nanogenerator by injecting a slippery lubricant into a porous surface (SLIPS) and combining it with transistor-inspired architectures. To enhance the solid–liquid triboelectrification performance and hydrophobicity of the coating, Wang et al. incorporated fluorine-containing materials into acrylic resin [60].

#### 2.2.2. Structural Optimization of the Solid–Liquid Triboelectric Nanogenerator

By manipulating the surface pattern of the system to alter the contact region between the friction pair, effective control over the triboelectric potential at the boundary can be achieved. Liu et al. [61] developed a bioinspired photoelectric–electromechanical merged TENG (Pem-iTENG) (Figure 4i). The improved efficiency was mostly due to the buildup of electrons via sunlight with negative triboelectric charges caused by touch electricity. Under tidal waves and sunlight, Pem-iTENG achieved a maximum open-circuit voltage of 124.2V and a peak power density of 172.3 W m^−2^, representing an almost tenfold increase compared to previously reported S-L TENGs utilizing solid–liquid contact electrification. While the output power was improved, the stability of outputs and the efficiency of energy transmission were also worthy of attention. Zhou et al. [62] successfully realized the volume effect by adding an end electrode to the traditional tube-based S-L TENG, and the output voltage was improved by about 40 times (Figure 4j). Sun et al. [63] constructed a new type of dual S-L TENG array. By assembling S-L TENGs in parallel, the total output performance could be highly improved, and more power could be provided for specific self-powered devices (Figure 4k). Li et al. [64] developed a droplet-based electricity generator incorporating a Kelvin water dropper (K-DEG) that could attain an ultra-high surface charge density of 358 μC m^−2^ within a short charging time of less than 1s (Figure 4l). The Kelvin water dropper could instantly inject abundant charges on the surface of the dielectric layer of the DEG as a result of corona discharge, while the DEG could fully release these surface charges into electricity generation upon droplet impinging. With such a high-density surface charge, the K-DEG could generate an enormously boosted transferred charge of 201 nC, output voltage up to 2000V, and instantaneous power density over 10^5^ W m^−2^ from one droplet impinging (100 μL).

## 3. Hydrovoltaic Effect

Water harbors immense energy in diverse forms, yet only a fraction of this potential has been effectively harnessed thus far. Nanostructured materials exhibit the ability to generate electricity through their interaction with water, a phenomenon referred to as the hydrovoltaic effect [5]. In comparison to bulk materials, nanomaterials exhibit exceptional sensitivity towards external stimuli and possess the capability to harness more diverse forms of water energy that are beyond the reach of conventional technologies. The rapid advancements in nanoscience and nanotechnology have significantly expanded the technical capabilities for harnessing mechanical and latent energies from water [65]. Over the past decade, there has been a significant surge in research endeavors focused on harnessing energy from water waves, raindrops, moisture, and evaporation through the utilization of nanomaterials [66,67,68,69]. However, a comprehensive understanding of hydrovoltaic mechanisms and enhancement in electrical outputs are still imperative for broader applications. The present section provides a tutorial review on diverse mechanisms for harnessing electricity from natural evaporation and ambient moisture, through an analysis of fundamental interactions occurring at various interfaces between water and materials.

### 3.1. Water–Solid Interfaces

**Figure 5:**
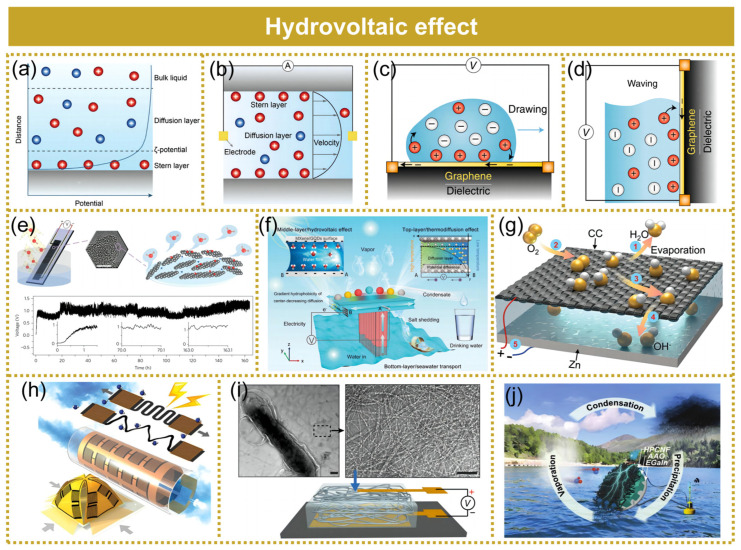
An overview of the mechanism of the hydrovoltaic effect and its power generation device. (**a**) Electric double layer at the interface between a solid and liquid. The blue curve illustrates the potential gradient close to the interface. (**b**) Diagram of the streaming potential in a nanochannel induced by a pressure gradient. The gray curve represents the velocity distribution of the nano-flow. Reproduced under the terms of the CC-BY license [65]. Copyright 2022, The Royal Society of Chemistry. (**c**) Depiction of the induced potential resulting from placing a droplet on graphene. An electric current is generated in graphene due to the movement of the double-layer boundaries at both the leading and trailing edges of the advancing droplet. (**d**) Schematic representation of the waving potential induced in graphene by a moving boundary of the double layer across a graphene layer placed on a dielectric substrate. Reproduced with permission [5]. Copyright 2018, Springer Nature. (**e**) Carbon black sheets capture the energy from evaporation occurring near the water’s edge, producing an open-circuit voltage that remains stable for 160 h in a normal environmental setting. Reproduced with permission [69]. Copyright 2017, Macmillan Publishers Limited, part of Springer Nature. (**f**) Schematic diagram of the working process of IEHVG. Schematic diagram of the structure and mechanism of the all-in-one IEHVG for sustained electricity output and water vapor under natural solar irradiation. Electrode selection A: Pt electrode, B: carbon nanotube (CNT) electrode. Reproduced with permission [78]. Copyright 2024, Wiley-VCH GmbH. (**g**) Schematic illustration of the device. Reproduced with permission [80]. Copyright 2023, Wiley-VCH GmbH. (**h**) Schematic drawing of GHEG preparation. Reproduced with permission [84]. Copyright 2018, WILEY-VCH Verlag GmbH & Co. KGaA, Weinheim. (**i**) Top, TEM images of the purified nanowire network (right panel) produced by the microorganism Geobacter sulfurreducens (dark shape in the left panel). Scale bars, 100 nm. Bottom, diagram of the device structure. Reproduced with permission [85]. Copyright 2020, Springer Nature. (**j**) Water cycle system for the MHEG. Reproduced with permission [88]. Copyright 2022, Wiley-VCH GmbH.

Upon contact between an aqueous solution and a charged solid surface, coulombic interactions give rise to the spontaneous formation of an electric double layer (EDL) consisting of a Stern layer and a diffusion layer (Figure 5a) [65]. The EDL creates a strong electric field, leading to a noticeable potential gradient across it. The boundary separating the Stern and diffusion layers is called the shear plane, defined by its zeta potential [70]. The Debye length is the separation from the shear plane to the nearest bulk liquid region, where the counter-ion density decreases as the distance from the shear plane increases.

**Streaming potential.** In 1859, Quincke observed that the application of a pressure gradient across a narrow channel induces the motion of electrolytes and generates a voltage in the fluid, known as the streaming potential [71]. When water is confined within a narrow region approaching the Debye length of the solution, the predominant occupancy in this space will be by counter-ions due to the overlapping of EDL (Figure 5b) [65]. Counter-ions are driven downstream by a pressure gradient, creating a flow that generates an electrical current until a stable voltage is reached within the channel, at which point the migration of ionic charges is halted [5]. The streaming potential induced in this manner depends on factors such as the pressure gradient, the geometry of the channels, and the hydrophilic characteristics of the surface. Flow-induced power generation utilizing nanomaterials, such as the graphene–water interface, has emerged as a compelling energy harvesting mechanism [72]. Jiao et al. [73] developed a flexible rope-like hydrovoltaic generator. The device demonstrated the potential for enhanced streaming potential by utilizing different electrolytes in a carbon black-based hyrovoltaic generator. The hydrovoltaic generator with a diameter of 5 mm and a length of 8 cm produced a maximum voltage of 0.66 V, a peak current of 873 μA, and a peak power density of 26.4 W m^−3^ by using 1.25 mol L^−1^ FeCl_3_ solution.

**Drawing potential.** In 2014, the application of carbon nanomaterials expanded the scope of streaming potential to encompass drawing and waving potentials. Yin et al. [67] demonstrated that the motion of an ionic solution droplet along a graphene strip could induce a voltage in the range of several millivolts. The drawing potential is generated by displacing a unique pseudocapacitor, which forms at the interface between the droplet and graphene, along the strip. When the droplet is propelled along the graphene surface, ions are adsorbed at the leading edge, propelling the pseudocapacitor forward and inducing electron migration within the graphene. Simultaneously, ions are desorbed at the trailing edge of the droplet, discharging the pseudocapacitor and releasing electrons into the graphene (Figure 5c) [5].

**Waving potential.** In comparison to droplets, waving water exhibits a higher abundance and possesses significantly greater energy potential, thereby enabling its harnessing through a methodology akin to that employed in the extraction of drawing potential. When a piece of graphene is submerged in an ionic solution or moves along the liquid–gas border, an electric double layer (EDL) forms at this dynamic contact, resulting in pseudocapacitance charging. Consequently, a voltage is induced within the graphene material (Figure 5d) [5]. The extraction of graphene from the water surface induces an inverse voltage as a result of the charge redistribution across the interface. Yin et al. recently observed this wave-induced voltage, which they referred to as waving potential [68]. The waving potential was found to be proportional to both the insertion velocity and sheet size. This waving potential was proportional to the inserting velocity and the sheet size. A 2 × 10 cm^2^-sized graphene sheet can produce a voltage of up to 0.1 V with a short-circuit current of 11 μA at a velocity of 1 ms^−1^. Scalability can be achieved through simple series or parallel connections of multiple graphene sheets. In addition to monolithic graphene-based devices, Fei et al. [74] demonstrated an alternative and efficient approach for electricity generation by utilizing two parallel graphene electrodes. An open-circuit voltage of up to 1 V was obtained as graphene moved across the electrolyte solution. In contrast to the generation of potential through wave propagation in a single graphene sheet, the potential in the two-graphene system was induced within the solution. In such a system, the moving graphene can be regarded as the driving force for ion motion, while the stationary graphene serves as a reference electrode. During the waving process, a dynamic electrochemical double layer (EDL) boundary is established, facilitating the continuous adsorption/desorption of cations on the graphene surface. This adsorption/desorption process generates a potential difference between the solution near the graphene surface and that in bulk.

### 3.2. Evaporation-Induced Electricity Generation

Water evaporation, an inherent and continuous process of phase transition from liquid to gas through the absorption of ambient heat, plays a pivotal role in sustaining the Earth’s hydrological cycle. Harvesting thermal energy from the natural evaporation process presents a promising approach for obtaining renewable and sustainable energy, aligning with the principles of nature conservation.

In 2017, Xue et al. developed a carbon-based device (Figure 5e), demonstrating the potential of utilizing water evaporation from various nanostructured carbon materials to generate electricity, which subsequently sparked significant research interest in subsequent years [69]. The device consisted of a porous carbon black (CB) layer, featuring multi-walled carbon nanotube electrodes at both ends. In standard conditions, the lower part of the device absorbed water, which then moved upwards through the CB film due to capillary action. Spontaneous evaporation at the exposed CB region facilitated sustained capillary movement over time. In this scenario, a CB-based device was able to continuously produce an electric potential close to 1.0 V (with a current reaching around 150 nA) between the two electrodes. Similarly, Ding et al. reported that a completely printed mesoporous carbon layer was capable of consistently producing a stable voltage of as high as 1 V, accompanied by a power density of approximately 8.1 W m^−3^ under ambient conditions through evaporation-driven water flow [75].

Evaporative hydrovoltaic generators have significant potential in addressing the water–energy crisis, but their applications are limited by low output voltage configurations resulting from slow phase transition rates of water molecules and intricate integration requirements. Li et al. presented an evaporation-driven water flow nanogenerator based on a piece of flexible carbon nanoparticle film [76]. The output of the generator was significantly enhanced to achieve a maximum open-circuit voltage of 5 V by synergistically combining modified carbon films with opposite surface charges. Shao et al. reported a bioinspired hierarchical porous fabric electrode that enabled a high water evaporation rate, efficient charge collection, and rapid charge transport in nanostructured silicon-based hydrovoltaic devices [77]. Recently, Chen et al. created a lotus-inspired inter-evaporation-driven hydrovoltaic generator (IEHVG) (Figure 5f), which efficiently generated steam and energy via the ocean rather than groundwater [78]. This innovative design achieved an exceptionally high voltage output exceeding the 100 V threshold through integrated forested IEHVG technology. Photothermal evaporation and electricity generation were highly enhanced by mimicking the transpiration process of a “stems-leaves of lotus”. At present, the main equipment for hydrovoltaic electrical energy output contains porous film with the bottom end immersed in a substantial fluid, while their present flow is restricted by pore passages [79]. Yu et al. [80] proposed the integration of capacitors and water evaporation devices to provide a stable power supply and presented a device with an asymmetric configuration made from water-attracting carbon fabric and a zinc plate (Figure 5g). The carriers generated through vaporization were possible carriers that were efficiently harnessed, resulting in a power density equal to 1.43 W m^−2^.

### 3.3. Moisture-Induced Electricity Generation

The presence of moisture, which is a combination of water vapor and air, represents one fundamental manifestation of water and encompasses an extensive reservoir of low-grade energy in the form of gaseous water molecules and droplets [81]. The abundance of moisture on Earth, amounting to 15 trillion liters, has spurred extensive research into moisture-induced electricity devices (MEDs) for the purpose of harnessing green and sustainable energy from this source [82]. There are two primary approaches to power generation. The first approach involves subjecting the entire device to moisture, enabling the generation of electric energy through ion gradient diffusion. The second method entails constructing an asymmetric structure in the hydrovoltaic device to generate electric energy. In this particular device, one side is encapsulated or coated with a water-absorbing material, while the other side is exposed to moisture [83].

Through a rational configuration design and with a versatile laser processing strategy, Yang et al. [84] achieved graphene-based hydroelectric generators (GHEGs) of sophisticated architectures, which had diversified functions such as rollable, stretchable, and even multidimensional transformation (Figure 5h). These graphene/hydrogel electrochemical generators (GHEGs) exhibited exceptional electricity-generation performance in both curled and elongated states, yielding voltages of up to 1.5 V in response to humidity fluctuations in the ambient atmosphere. Liu et al. [85] fabricated a moisture electric generator from a thin film of protein nanowires (Figure 5i). The device produced a sustained voltage of around 0.5 volts across a 7-micrometre-thick film, with a current density of around 17 microamperes per square centimeter. Liu et al. [86] reported a moist electric generator (PN-MEG) using peptide nanofibrils from milk β-lactoglobulin. The created gadget could produce an open-circuit voltage of as high as 0.65 V, a short current of 2.9 µA, and an absolute maximum power consumption of 0.39 W m^−2^. This device’s outstanding performance was due to its outstanding hydrophilicity, rapid ionization, and surface-to-volume proportion of protein nanofibrils. Density functional theory (DFT) studies revealed that the carboxyl functional groups in amino acid chains inside protein nanofibrils functioned as active sites for water molecules to undergo binding and ionization. By employing HCl/polyvinyl alcohol (PVA) electrolyte gel to bridge two carbon nanotube (CNT) electrodes, and incorporating CaCl_2_ on one side, Luo et al. [87] successfully fabricated a novel moisture-induced self-charging device (MISD). The inclusion of a widely used and cost-effective desiccant, CaCl_2_, in one electrode enabled effective water absorption from the surroundings. The carbon nanofiber electrode designed by Lu et al. [88] exhibited a hierarchical porous framework that allowed for quick penetration and diffusion of water molecules (Figure 5j). This electrode acts like a nanofluidic diode by employing anodic aluminum oxide as its material. The intrinsic magnetic field drives the selective and directed movement of ions while efficiently converting ion/electron currents at both electrodes.

### 3.4. Material Classification

**Figure 6:**
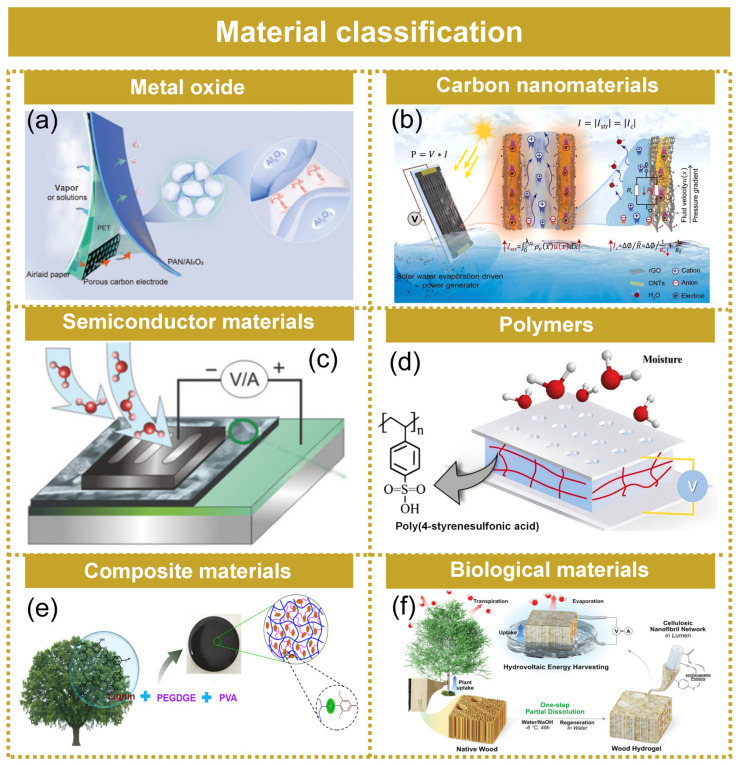
Material classification of hydrovoltaic technologies. (**a**) Metal oxide materials: schematic diagram of a hydrovoltaic device based on a flexible tough PAN/Al_2_O_3_. Reprinted by license [89]. Copyright 2023, Wiley-VCH GmbH. (**b**) Carbon nanomaterials: illustrative representation of the porous rGO/CNT film. Reproduced under the terms of the CC-BY license [90]. Copyright 2023, Wiley-VCH GmbH. (**c**) Semiconductor materials: schematic diagram of a hydrovoltaic device based on TiO_2_ nanowires. Reproduced with permission [91]. Copyright 2018, Wiley-VCH. (**d**) Polymers: schematic diagram of a hydrovoltaic device based on a PSSA membrane. Reproduced with permission [92]. Copyright 2019, Royal Society of Chemistry. (**e**) Composite materials: schematic diagram of hydrovoltaic device based on PVA/lignin composite material. Reprinted by license [93]. Copyright 2021, Elsevier B.V. (**f**) Biological materials: illustrative representation of the hydrovoltaic device based on wood. Reproduced with permission [94]. Copyright 2022, Wiley-VCH GmbH.

With recent advances in nanotechnology, many nanomaterials have now been developed to generate attractive electricity output (Figure 6). According to the morphology and output characteristics of the materials, hydrovoltaic materials are classified into six categories: metal oxides, carbon nanomaterials, polymer materials, semiconductor materials, biological materials, and composite materials.

In 2023, Li et al. [89] developed a flexible tough polyacrylonitrile/alumina (PAN/Al_2_O_3_) hydrovoltaic coating with both good electricity generation (open-circuit voltage, Voc ≈ 3.18 V) and sensitive ion sensing (2285 V M^−1^ for NaCl solutions in 10^−4^ to 10^−3^ m) capabilities (Figure 6a). The porous nanostructure composed of Al_2_O_3_ nanoparticles was firmly locked by the strong binding effect of PAN, giving a critical binding force 4 times that of Al_2_O_3_ film to easily deal with 9.92 m s^−1^ strong water-flow impact. High electrical conductivity, simple preparation, and low cost enable carbon nanomaterials to be one prevalent material. Wu et al. [90] fabricated an efficient water evaporation-driven EPG based on porous reduced graphene/carbon nanotube film (rGO/CNT) that enabled a high water evaporation rate and high electrical conductivity through 3D printing technology (Figure 6b). The high evaporation rate and high electrical conductivity promoted ion migration in channels and charge transport on the rGO layer, boosting water evaporation-driven device performance. Semiconductor materials have the advantages of strong mechanical fastness and stable chemical properties, so they have attracted much attention in the field of hydrovoltaic technology. Zhou et al. [91] synthesized a titanium dioxide nanowire (TDNN) with a negative charge, and the nanowire networks contained an abundance of interstitial nanochannels (Figure 6c). Electricity was generated from the diffusion of ions through the many 3D nanochannels in the TDNN. At a relative humidity of 85%, the output power density exceeded ≈4 µW cm^−2^ from a 1.2 × 1.2 cm device, which was three orders of magnitude better than that from monolayer graphene or polypyrrole generators. Polymers are made from repeated geometric monomer units. Single molecules of a polymer are made up of a vast number of monomer components, typically with a straight-ahead, branching, or network-like structure. Polymers like these include a range of sulfate or functional groups with oxygen (such as hydroxyl, carboxylic groups, and sulfonic acid group) that liberate protons when they are brought into contact with water molecules. A 1 cm^2^ PSSA membrane can provide an open-circuit voltage of 0.8 V and a short-circuit current density of 0.1 mA cm^−2^, enough for powering numerous electronic devices (Figure 6d) [92]. Composite materials typically preserve the structural features of the primary constituents, while the incorporated additives impart additional attributes or functional surfaces to boost electrical performance. Zhang et al. [93] used lignin to improve strength and impart pH-responsive properties of PVA hydrogel (Figure 6e). The lignin-reinforced PVA (LRP) hydrogel had a maximum storage modulus of 83.1 kPa, which was much higher than the PVA hydrogel. Biological materials from renewable resources have recently attracted more attention because of their biodegradable and biocompatible nature. The development of bio-based hydrovoltaic materials advances sustainability further into the future, with wood being a promising candidate due to its innate hydrophilic and anisotropic composition. Jonas et al. [94] demonstrated an efficient hydrovoltaic wood power generation using wood cell walls with nanotechnology (Figure 6f). A highly permeable wood with a fiber network covering the lumen was created utilizing an environmentally friendly, single-step treatment with sodium hydroxide to increase wood surface area, add chemical functionality, and improve water permeability in the cell wall.

## 4. Nanomaterial Selection

The rapid development of nanotechnology and nanomaterials has greatly expanded the technical capabilities of harnessing the mechanical and potential energy of water. Table 1 shows the nanomaterial engineering of solid–liquid nanogenerators in recent years. In 2020, Jiang et al. [95] developed an effective method to fabricate stretchable electrodes by coating MXene ink on an elastic fiber and then spontaneously growing silver nanoparticles (AgNPs). After coating MXene and the growth of AgNPs, a highly stretchable triboelectric yarn was obtained. Electrospinning is a classic nanofiber preparation method; the surface morphology with micro/nanopatterns and high mechanical flexibility endowed by electrospinning increases the effective contact area between the triboelectric active layers and improves the output performance [96,97]. Li et al. prepared janus-type thermoplastic polyurethane (TPU) nanofiber films by electrospinning [98]. The hydrophobicity of the TPU and the output performance of the corresponding S-L TENG were improved. The addition of Ga to the enclosed S-L TENG increased its output performance to reach a peak voltage of 282 V. Compared with an S-L TENG based on a nanofiber network, an S-L TENG based on a vertically aligned nanowire structure usually has a higher performance enhancement factor, which may be due to a higher specific surface area and more flexible contact mode. Kim et al. used plasma etching to etch nanowire arrays on the surface of a glass substrate and then covered them with polystyrene nanoparticles to obtain moth eye-inspired substrates and triboelectric layer surfaces [99]. It had excellent light transmittance, which made the water-based TENG closer to the excellent complementary energy harvester of solar cells to achieve simultaneous collection of solar energy and raindrop energy.

Li et al. demonstrated a solar thermal conversion-boosted hydrovoltaic power generation system (HPGS) [100]. In the system, the Al_2_O_3_/CB nanoparticle-constructed hydrovoltaic functional coating exhibited remarkable solar thermal conversion ability. Under 1 standard solar radiation, the device temperatures increased by more than 18 °C, and the V_oc_ increased from 2.54 V to 5.86 V, an increase of 130.7%. By repeatedly dipping silk fibroin (SF) on electrospun nylon-66 nanofiber (NNF) films, zhang et al. achieved surface polarity enhancement, precise fiber size control (≈25 nm), and strong nanostructure bonding [101]. The open-circuit voltage of the flexible self-supporting water voltaic device prepared in deionized water was as high as 4.82 V. Duan et al. developed an efficient organic–inorganic hybrid MEG by in situ preparation of highly hygroscopic polyacrylamide ionic hydrogel on silicon nanowire arrays [102]. The robust silicon nanowires had abundant cation-selective nanochannels, thereby significantly improving the electrical output of MEGs.

## 5. Reverse Electrowetting

**Figure 7:**
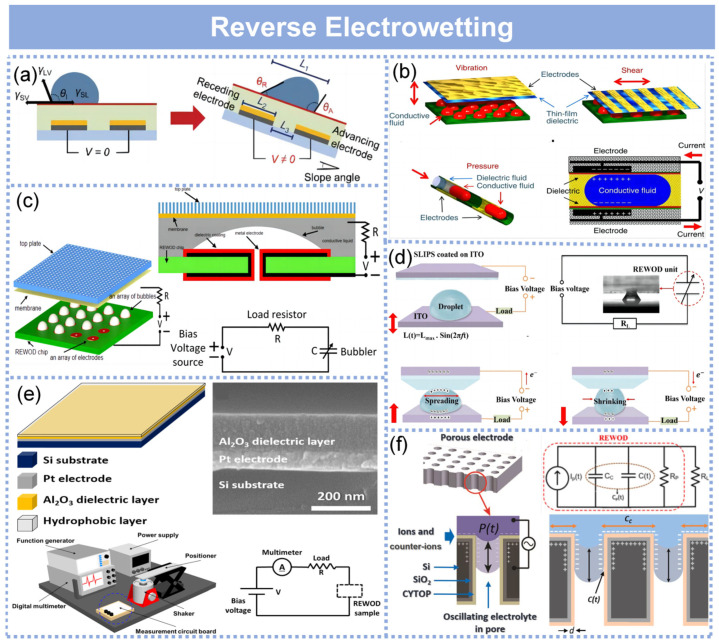
An overview of the mechanism of reverse electrowetting and energy harvesting based on reverse electrowetting. (**a**) Schematic of the energy harvesting mechanism based on the reverse phenomenon of REWOD. By tilting the chip, the contact angle hysteresis of a sliding water droplet generates a relative voltage difference between the receding electrode and the advancing electrode. Reproduced with permission [103]. Copyright 2021, Wiley-VCH GmbH. (**b**) Schematics for three primary droplet actuation systems. The top panel shows particles between oscillating slabs (left) and droplets between sliding slabs (right). The bottom panel depicts droplets in a tiny channel (left) and schematics of reverse electrowetting-based energy production in a microchannel architecture (right). Reproduced under the terms of the CC-BY license [15]. Copyright 2011, Macmillan Publishers Limited. (**c**) The bubbler concept. Left panel: Schematics of a bubbler device. Right panel: Cross-sectional view of a single bubble (top) and an equivalent electrical circuit that corresponds to the bubbler device (bottom). Reproduced with permission [105]. Copyright 2015, Springer Nature. (**d**) The schematic diagram and circuit model of REWOD-DEG. Reproduced with permission [106]. Copyright 2023, Royal Society of Chemistry. (**e**) Top panel: schematic of the REWOD energy harvesting cell (left) and a cross-sectional image of the fabricated sample captured by FE-SEM (right). Bottom panel: schematic diagrams of the home-made measurement station (left) and the measurement circuit (right). Reproduced with permission [107]. Copyright 2016, Elsevier Ltd. (**f**) REWOD energy harvesting. Left panel: the working principle of reverse electrowetting power generation with a high surface area, driven by pulsating pressure, P(t). Right panel: the RC model of the REWOD energy harvester, connected in parallel to a load resistor RL (top), along with a schematic diagram highlighting the regions on the porous electrode responsible for generating CC and C(t) (bottom) [25]. Copyright 2021, Elsevier B.V.

Electrowetting can be regarded as the conversion of electrical energy into mechanical energy, with a reversible process enabling mechanical-to-electrical energy conversion. A droplet is periodically deformed by an external mechanical force, thereby inducing a change in the contact area between the droplet and the dielectric-coated electrode connected to the bias voltage source (Figure 7a) [103]. The periodic variation in the contact area induces a corresponding change in the electrical capacitance of the interface, resulting in an oscillating flow of electrical current across the load resistor and generating energy [14]. This novel phenomenon, called reverse electrowetting on dielectric (REWOD), was described by Krupenkin and Taylor [15]. The primary objective of the bias voltage source in the REWOD process is to induce an adequate charge density at the solid–liquid interface. The bias voltage source does not contribute to any network generation during the energy production process [104]. In the REWOD process, fluidic actuation can be accomplished in various geometries, as illustrated in Figure 7b, including out-of-plane vibration, in-plane shear, and droplet motion within channels [15]. To further elucidate the fundamental principle of REWOD operation, let us consider the case of a droplet confined within a channel, as depicted in Figure 7b (left). As the droplet undergoes alignment and misalignment with the electrified thin film dielectric-coated electrodes, variations in electrical charge at the electrodes result in consequential changes, thereby inducing an electric current flow, as illustrated in Figure 7b (right).

Power generated using REWOD can be maximized by two primary approaches. Firstly, by enhancing the energy produced during each droplet oscillation, particularly by increasing the applied bias voltage or augmenting the capacitance of the solid–liquid interface. However, this approach is subject to an evident limitation since a higher capacitance necessitates a thinner dielectric film, thereby imposing restrictions on the maximum bias voltage that can be applied across the interface without encountering the risk of electrical breakdown. The second approach involves increasing the oscillation frequency of the droplet, thereby augmenting both the overall power output through a higher rate of energy generation events and the energy yield per oscillation due to the intricate dynamics of electrical charge transfer during the REWOD process. Tsung et al. [105] developed a novel technique for converting mechanical energy directly into electrical energy through microfluidic systems. This approach integrates the previously observed reverse electrowetting on dielectric (REWOD) process with the fast oscillating behavior of bubble formation and collapse. By combining the rapid dynamics of bubbles with REWOD, the electrical power generated can be increased by more than an order of magnitude compared to using REWOD alone. This technique is well suited for extracting energy from various mechanical sources characterized by a wide range of frequencies, enabling a significant enhancement in generated power proportional to the product of oscillation frequency and energy produced during each oscillation. Cheng et al. [106] innovatively introduced a slippery lubricant-infused porous surface (SLIPS) into a reversed-electrowetting-based droplet electricity generator (REWOD-DEG), which had low contact angle hysteresis and low charge trapping characteristics and was capable of stable power generation for a long time at high bias voltage (Figure 7d). Based on the vibrating plate-type REWOD-DEG, adjusting the conductive droplet concentration, vibration frequency, and vibration amplitude to regulate the output power, the best matching load of the system was obtained, and the maximum output power of the generator was 143 nW. When exposed to a variety of extreme operating conditions, the device showed robust performance.

REWOD generates electricity from the deformation of fluid droplets induced by mechanical energy. When mechanical energy, like a vibration, is applied to a system, the contact area is deformed and the amount of charge at that area is altered. The amounts of charges at the contact area are decided by the capacitance of the area. Thus, in REWOD, the mechanical energy of liquid motion is converted to electrical energy, which is extensively based on the capacitance of the dielectric layer. The high capacitance of a dielectric layer can be obtained by (a) the use of a high-k material (a material with a high dielectric constant) and (b) thin as well as dense film fabrication. In that context, a high capacitance, high-k material is adopted and a high-quality thin film of the same is prepared via atomic layer deposition (ALD), which is a state-of-the-art technology for thin film fabrication without defects. Yang et al. [107] successfully created extremely thin Al_2_O_3_ layers of approximately 100 nm thickness using atomic layer deposition (ALD), resulting in solid films with significantly enhanced capacitance. When comparing ALD films with sputtered thin films, ALD films exhibited higher capacitances and lower leakage current densities (Figure 7e). The performance of REWOD with ALD Al_2_O_3_ films was excellent at low voltage and excitation frequencies, achieving a maximum power density of 110 W/m^2^ with a DC bias of 24 V and an external excitation frequency of 2 Hz. Later, Yang et al. [108] explored the use of high-k dielectric material TiO_2_ in combination with a novel Al_2_O_3_ leaky barrier layer. This layered structure demonstrated reduced current leakage and higher capacitance compared to single-layer TiO_2_ and Al_2_O_3_, thus enhancing power output.

In REWOD, the generation of current is influenced by the contact area between the electrodes and the electrolyte. One way to enhance this interfacial area is by increasing the number of electrolyte droplets [109]. However, improving the current output by enlarging the contact area per unit electrode area cannot be achieved solely by adding more droplets. Another method is to use porous materials with a high pore depth-to-diameter ratio. P.R. et al. [25] introduced a novel approach to improve REWOD energy harvesting by significantly increasing the surface area through the incorporation of perforated silicon wafer electrodes. Figure 7f illustrates the working principle of high-surface-area REWOD energy harvesting, where alternating current is generated by the movement of a liquid electrolyte through micro-pores under pulsating pressure. The system operates at a given frequency, causing the liquid electrolyte to oscillate in and out of the micro-pores, changing the electrode–electrolyte interface and generating alternating current. Without the application of an external bias, the peak current and voltage densities were 3.77 μA/cm^2^ and 1.05 V/cm^2^ at a modulation frequency of 5 Hz, with a power density of 0.048 μW/cm^2^. This is approximately 23 times higher than the output obtained using planar electrodes in previous studies, demonstrating the critical role of porous electrodes in improving REWOD energy harvesting performance. The innovation of this work lies in its bias-free approach for energy harvesting, combined with the significant increase in electrode surface area, resulting in a substantial boost in power generation efficiency.

## 6. Tribovoltaic Effect

Tribovoltaic action arises from the creation of electron–hole pairs at the interface of the PN junction, which occurs due to the energy released during the formation of new chemical bonds through mechanical sliding. Furthermore, an internal electric field at the junction efficiently separates the charge carriers, generating a direct current (DC) output. The energy generated during the formation of a chemical bond is referred to as “bindington”, which serves as the excitation to stimulate electron–hole pairs, much like the mechanism seen in photovoltaic action [110,111,112,113]. The only difference between the two is the source of energy that excites the electron–hole pairs [110]. The definition of the “tribovoltaic effect” was proposed firstly by Wang and Wang in 2019 [113] and subsequently mentioned by Z. Zhang et al. in 2020 in an experimental study on the relative sliding of metal onto a silicon surface [111]. Similar to contact electrification, the tribovoltaic effect not only occurs at the solid–solid interface but also at the solid–liquid interface.

### 6.1. Liquid–Semiconductor Interface

**Figure 8:**
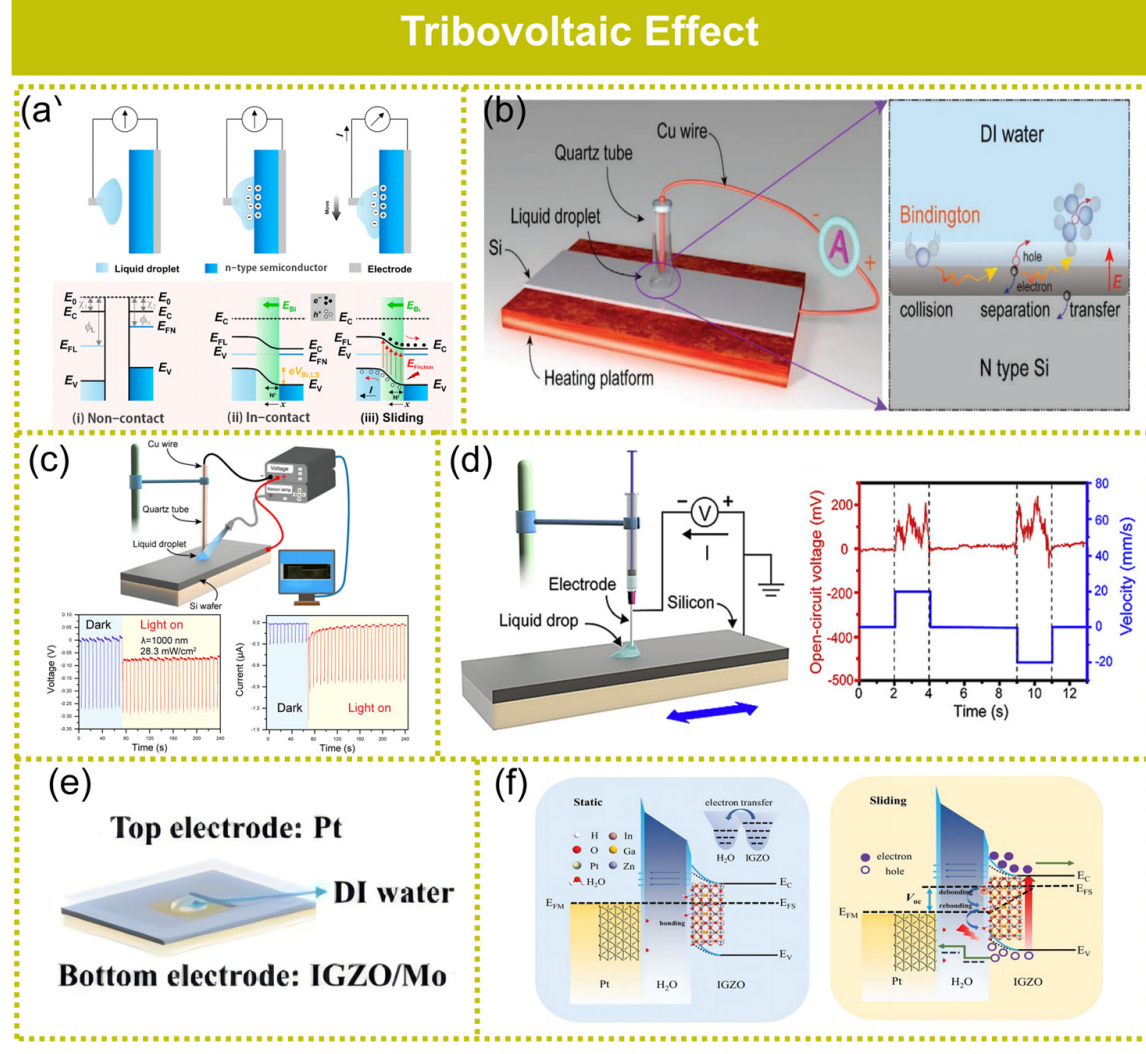
Tribovoltaic effect at the solid–liquid interface. (**a**) Working principle (top) and energy band diagram of the LS junction in non-contact, in-contact, and sliding states (bottom) of the LS junction-based TVNG. Reproduced with permission [16]. Copyright 2023, Elsevier Ltd. (**b**) Effect of temperature on the tribovoltaic effect at the DI water and silicon interface. Reproduced under the terms of the CC-BY license [120]. Copyright 2021, Wiley-VCH GmbH. (**c**) Effect of light irradiation on the tribovoltaic effect at the DI water and silicon interface. Reproduced with permission [121]. Copyright 2021, Elsevier Ltd. (**d**) Effect of droplet sliding speed on the tribovoltaic effect at the DI water and N-type Si interface. Reproduced with permission [24]. Copyright 2020, Elsevier Ltd. (**e**) Schematic illustration of the FLG. (**f**) Left panel: The diagram illustrating the spectrum composition and the dynamics of charge carriers in the stationary Pt/H2O/IGZO structure within the FLG is provided. Right panel: The schematic of the frequency structure and the carrier dynamics of the fluid Pt/H2O/IGZO structure in FLG during sliding is presented. This material was reproduced with permission [108], Copyright 2022, Wiley-VCH GmbH.

Certain physical phenomena at the solid–liquid interface can be employed to elucidate the charge generation mechanism for LS junction. As depicted in Figure 8a, when a liquid comes into contact with the surface of a semiconductor (e.g., n-type material), it induces charged surfaces proximal to the interface. In a typical solid–liquid interface, surface ionization reactions occur, resulting in the formation of an extremely thin electrical double layer (EDL) serving as a junction field [113]. During contact, charges are transferred between solid atoms and water molecules through the overlap of electron clouds [114]. The contact electrification of liquids (e.g., water) and semiconductors results in the generation of charged surfaces. The difference in Fermi levels between the two materials (Figure 8a(i)) leads to the creation of a built-in electric field (Figure 8a(ii)). Triboelectrification occurs, generating charges that are subsequently swept out by the electric field into the external circuit. The number of transferred charges is enhanced when a high level of frictional energy (E_Friction_) is applied (Figure 8a(iii)).

### 6.2. Influencing Factors of the Solid–Liquid Tribovoltaic Effect

Temperature significantly influences the energy band structure and surface characteristics of semiconductors, a topic extensively investigated in photovoltaic [115,116] and catalysis studies [117,118]. It is widely acknowledged that the reaction rate at the solid–liquid interface is significantly influenced by temperature, thereby emphasizing its pivotal role in governing the bonding kinetics at this interface [119]. According to the concept of “bindington”, it is anticipated that temperature will exert a significant influence on the rate of “bindington” formation, subsequently impacting the generation of tribo-voltage and tribo-current at the liquid–semiconductor interface. Zheng et al. [120] investigated the impact of temperature on the tribovoltaic effect at solid–liquid interfaces while also demonstrating the synergistic influence of liquid pH value and temperature on the tribovoltaic effect (Figure 8b). The results demonstrated that an increase in temperature led to a corresponding rise in tribo-voltage and tribo-current at the water/Si and water/metal interfaces during sliding. Elevated OH^−^ concentrations and higher temperatures exacerbated the generation rate of “bindington” and resulted in a greater release of thermal energy through redox reactions, thereby increasing the intrinsic carrier concentration, which subsequently impacted the electrical output of the tribovoltaic effect. Zheng et al. [121] employed a quartz tube to facilitate the movement of a water droplet across both N-type and P-type Si surfaces (Figure 8c) under varying light irradiation conditions. The findings revealed that the tribo-current exhibited consistent directionality with the photo-current, while light irradiation was observed to augment the tribo-current at the liquid–semiconductor interface. Moreover, this enhancement in the tribo-current intensified with increasing light intensity and decreasing light wavelength. Lin et al. [24] employed an electrode probe to facilitate the controlled sliding of a water droplet on a silicon surface (Figure 8d). This experimental setup resulted in the generation of both tribo-voltage and tribo-current, allowing for further investigation into the influence of droplet sliding speed and static contact area on the observed tribovoltaic effect. The results demonstrated that the voltage and current exhibited an increasing trend with the sliding speed. Notably, the voltage remained unaffected by the static contact diameter of the droplet. Conversely, the current displayed a positive correlation with the contact area.

### 6.3. Solid–Liquid Tribovoltaic Nanogenerator

Huang et al. [122] proposed a flexible liquid-based DC generator (FLG) with outstanding safety and performance, as shown in Figure 8e. In this design, the semiconductor layer is an indium gallium zinc oxide (IGZO) film, known for its excellent film consistency, mechanical flexibility, and stable properties. The generator is sealed with polydimethylsiloxane (PDMS) for protection. The FLG forms a solid–liquid–solid sandwich structure, where the metal platinum electrode and IGZO semiconductor layer are connected through an intermediate liquid medium. Charge transfer occurs when liquid molecules adsorb and bind to the solid surface. In thermal equilibrium, the system reaches a unified Fermi level and no additional current flows into the system. The difference in work function between platinum (ΦPt ≈ 5.65 eV) and indium gallium zinc oxide (IGZO, ΦIGZO ≈ 4.50 eV) causes the energy bands of the semiconductor to bend, resulting in the formation of a space charge region and an electric field, similar to a PN junction, as shown in Figure 8f (left). When the liquid insertion medium slides along the surface of the semiconductor, mechanical energy causes the separation of previously bonded liquid molecules from the semiconductor, leading to the release of energy. This released energy is sufficient to excite excess electron–hole pairs in the space charge region of the semiconductor, which are separated by the internal electric field. Since the field direction is from IGZO to Pt, the holes drift towards the Pt electrode, while the electrons move towards IGZO, creating a non-equilibrium condition (Figure 8f, right). When the FLG is slightly shaken or tilted, a short-circuit current of 3.5 µA and an open-circuit voltage of 620 mV are generated.

## 7. Applications

**Figure 9:**
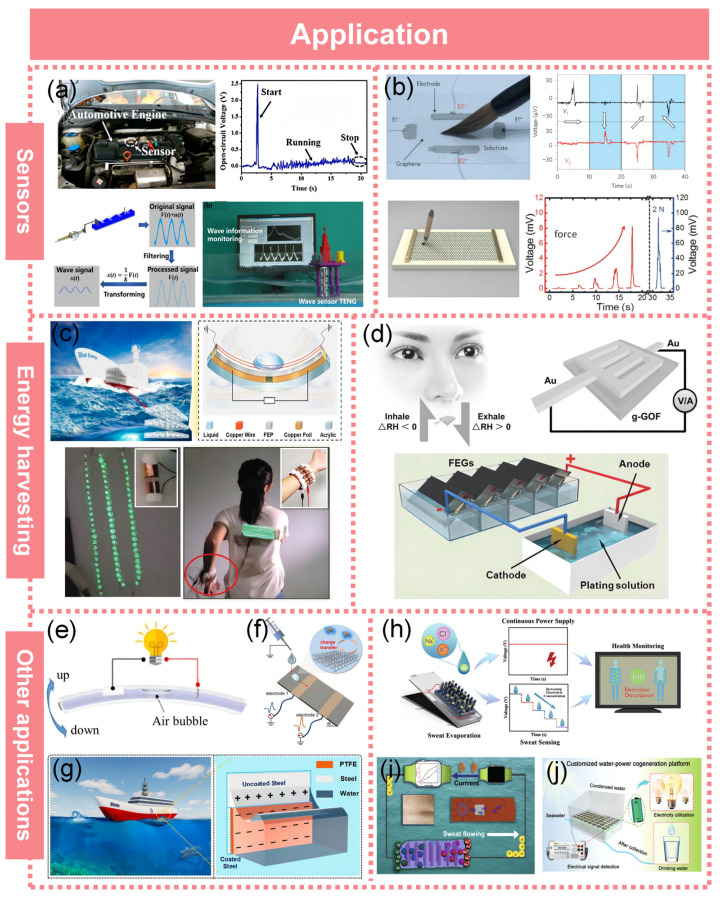
Typical applications of solid–liquid TENGs and water-induced electricity devices. (**a**) Top panel: demonstration of the acceleration sensor for vibration monitoring. The acceleration sensor was fixed onto the automotive engine to monitor their working state. Bottom: the process of wave monitoring signals obtained from the WS-TENG and the WS-TENG applied to monitor waves around the leg of a marine platform. Reproduced with permission [126]. Copyright 2017, American Chemical Society. Reproduced with permission [127]. Copyright 2018, Elsevier Ltd. (**b**) Top panel: handwriting with a Chinese brush on graphene and sensing the stroke directions (arrows) by the drawing potentials between electrodes E1^+^-E1^−^ and E2^+^-E2^−^. Bottom panel: schematic diagram of measuring the electrical response when we use a Chinese brush to increase the pressure applied on the substrate and typical voltage responses as we gradually increase the force applied to the substrate. Reproduced under the terms of the CC-BY license [67]. Copyright 2014, Springer Nature; Reproduced under the terms of the CC-BY license [113]. Copyright 2016, WILEY-VCH Verlag GmbH & Co. KGaA, Weinheim. (**c**) Top panel: structural diagram of DB-TENG arrays on the deck of a model ship of Blue Energy. Bottom panel: 150 LEDs being powered by swing arms wearing a WT-TENG wristband with 10 units. The red circle labels the position of the WT-TENG wristband. The inset shows the WT-TENG wristband. Reproduced with permission [18]. Copyright 2021, American Chemical Society. Reproduced with permission [131]. Copyright 2021, Wiley-VCH GmbH. (**d**) Top panel: harvesting the body energy hidden in the respiratory moisture tide. Bottom panel: schematic diagram of the setup for electrodeposition. Reproduced with permission [66]. Copyright 2015, WILEY-VCH Verlag GmbH & Co. KGaA, Weinheim. Reproduced with permission [75]. Copyright 2017, WILEY-VCH Verlag GmbH & Co. KGaA, Weinheim. (**e**) The B-TENG as a water level monitoring system to detect the water level height. Reproduced with permission [133]. Copyright 2022, Elsevier Ltd. (**f**) When a drop of liquid flows through the polymer surface, the charge transfer between liquid and solid occurred, and the current signals were measured by the two Cu electrodes separately. Reproduced with permission [134]. Copyright 2021, American Chemical Society. (**g**) Device configuration of the SL-TENG installed on the hull and schematic representation structure for the SL-TENG. Reproduced with permission [22]. Copyright 2022, American Chemical Society. (**h**) Schematic diagram of self-powered sweat analysis and its application in health monitoring. Reproduced with permission [135]. Copyright 2023, Wiley-VCH GmbH. (**i**) Optical image of the wearable battery-free perspiration analyzing sites. Reproduced with permission [136]. Copyright 2020, Springer Nature. (**j**) Self-designed outdoor freshwater–electricity cogeneration system. Reproduced with permission [78]. Copyright 2024, Wiley-VCH GmbH.

The development of various water-based electricity devices (WEDs) has resulted in the emergence of uses such as energy generators and detectors, as shown in Figure 9.

### 7.1. Sensors

Under external mechanical stimulation, an S-L TENG can generate current and voltage signals, enabling its utilization as a self-powered sensor for the characterization of various mechanical stimuli, including vibration, sound, and impact. Herein, we present illustrative examples to demonstrate the diverse applications of self-powered sensors across multiple disciplines. Chen et al. [123] introduced a self-powered triboelectric microfluidic sensor (TMS) that utilizes signals generated by droplets and bubbles through capillary forces and triboelectrification at the liquid–solid interface, enabling the immediate detection of liquid and gas movement. The fabrication of a superhydrophobic solid–liquid contact triboelectric nanogenerator and its biomedical applications as droplet sensors were reported by Hu et al. [124]. Zhang et al. [125] developed a smart U-shaped water–solid triboelectric nanogenerator by utilizing the solid–liquid triboelectrification of water in a U-tube, enabling self-powered pressure and mechanical motion sensing capabilities. The U-shaped triboelectric nanogenerator exhibited remarkable sensitivity as a self-powered displacement sensor with values of 0.91 V mm^−1^ and 8.50 nA mm^−1^, respectively. Additionally, it demonstrated high sensitivity as a pressure sensor with values of 4.41 V kPa^−1^ and 72.94 nA kPa^−1^. Zhang et al. [126] presented a self-powered and highly sensitive acceleration sensor based on an S-L TENG. This acceleration sensor exhibited an extensive detection range of 0 to 60 ms^−2^ and achieved a remarkable sensitivity of 0.26 V·sm^−2^ (Figure 9a, top). The highly sensitive wave sensor based on a solid–liquid interfacing triboelectric nanogenerator was proposed and systematically investigated by Xu et al. It was observed that the output voltage exhibited a linear increase with wave height, demonstrating a sensitivity of 23.5 mV mm^−1^ for an electrode width of 10 mm. These findings indicate that the wave sensor is capable of accurately detecting wave heights in the millimeter range (Figure 9a, bottom) [127].

Yin et al. [67] employed a square graphene sheet as a medium for handwriting detection, utilizing a Chinese brush immersed in a 0.01 M NaCl solution. To discern the direction of handwriting, two sets of electrodes (E1^+^-E2^−^ and E2^+^-E2^−^) were strategically patterned orthogonally along the four edges of the graphene substrate (Figure 9b, top). Zhong et al. [113] used a graphene–PVDF piezoelectric film structure as a 2D force sensor by using a Chinese brush dipped in DI water to change the pressure applied on the substrate, as shown in Figure 9b, bottom left. As we can see from Figure 9b, bottom right, we can obtain a higher voltage output with a stronger force applied to the substrate. When the brush slightly sweeps over the graphene surface, a peak voltage around 0.2 mV can be generated. As we gradually increase the force applied to the substrate, the voltage increases to ≈8.2 mV, and a voltage output up to 0.1 V can be generated with a ≈2 N force.

### 7.2. Energy Harvesting

Harnessing the vast potential of oceanic resources, such as wave energy, presents a significant opportunity for solid–liquid generators to make a substantial contribution towards global energy requirements. Li et al. [9] designed a solid–liquid contact buoy triboelectric nanogenerator, which exhibited a buoy-like structure and enabled efficient energy harvesting from various low-frequency vibrations, including up-down, shaking, and rotational movements. By establishing a network of these buoy S-L TENGs, significant amounts of energy can be harvested from surface waves and submarine currents to power portable electronic devices or navigation systems. Recently, a fully enclosed iTEHG based on a triboelectric nanogenerator and electromagnetic generator (EMG) was proposed, with the structure design of a unique concentric circular electrode pair for efficient and sustainable harvesting of omnidirectional ocean wave energy [128]. The iTEHG produces unprecedented performance with 360-degree undifferentiated large-magnitude power and current densities of 7.25 W·m^−3^ and 4.85 A·m^−3^ with less than 5% relative standard deviation between different directions. The droplet-based triboelectric nanogenerator (DB-TENG) has a simple open structure, and the integrated DB-TENG network array is of great significance for all-weather ocean energy harvesting (Figure 9c, top) [18]. Under a simulated ocean wave, a nonpackaged scaled-up DB-TENG with considerable output performance can charge capacitors or drive electronic devices. Li et al. [129] proposed a droplet-based electricity generator with a simplified open structure (SCE-DEG) that effectively harnesses the self-capacitance effect of the upper electrode. This innovative design serves as a valuable reference for large-scale raindrop energy harvesting. Zhang et al. [130] proposed a fluorinated superhydrophobic greenhouse film as a negative triboelectric layer material for the construction of a raindrop energy harvesting triboelectric nanogenerator (RDE-TENG). Wu et al. developed a versatile and high-performance water tube-based TENG (WT-TENG) by encapsulating deionized DI water into a fluorinated ethylene–propylene tube. Furthermore, they fabricated a portable device in the shape of a wristband integrated with 10 identical small WT-TENG units (Figure 9c, bottom) [131]. By wearing this WT-TENG wristband, it was demonstrated that 150 LEDs could be powered through arm swinging or shaking.

Due to their affordability, flexibility, and high energy output, WEDs are easily adaptable and can be combined to improve everyday human activities. In 2015, Zhao et al. [66] developed a g-GOF-based power generator for sensitive harvesting of breath energy (Figure 9d, top). The respiration of a healthy individual (ΔRH = 21%) can generate an output voltage and current of approximately 18 mV and 5.7 µA cm^−2^, respectively. This power generator has the potential to serve as a respiratory energy harvester for converting bodily energy into electrical power. The continuous and stable electricity generated from water evaporation shows great potential for practical applications in energy harvesting systems. For instance, a series connection of five CB-based units generated an open-circuit voltage of 1.45 V and a short-circuit current of 2.85 mA, which was sufficient to facilitate silver electrodeposition at the micrometer scale (Figure 9d, bottom) [75]. Similarly, Zhou et al. [132] utilized the anisotropic three-dimensional structure of wood to fabricate nanogenerators that harness streaming potential/current for electricity generation. By connecting five wood nanogenerators in series, a functional calculator was demonstrated as a practical application.

### 7.3. Other Applications

Li et al. demonstrated a newly designed bubble-based solid–liquid triboelectric nanogenerator that operates by moving air bubbles within a liquid-filled PTFE tube. This system was integrated into a water level monitoring setup to measure water height (Figure 9e) [133]. Zhang et al. [134] explored the charge transfer process between liquid and solid interfaces using a novel self-powered droplet triboelectric nanogenerator, which is equipped with spatially arranged electrodes serving as probes (Figure 9f). Another promising application of nanogenerators is self-powered corrosion protection and antifouling. Effective corrosion protection of metals is crucial in marine engineering. Zhang et al. [22] reported an SL-TENG, where a dielectric film serves as the organic coating, and both coated and uncoated steel electrodes in seawater exhibit marine corrosion resistance (Figure 9g).

Human sweat comprises various electrolytes that are health status indicators. A hydrovoltaic device was successfully fabricated by Chen et al. using silicon nanowire (SiNW) arrays incorporated with modified carbon nanoparticles [135], as illustrated in Figure 9h. This innovative electric nanogenerator exhibited the remarkable capability of electrolyte sensing in human sweat. The continuous generation of open-circuit voltage and short-circuit current was achieved by exploiting water flow within the nanochannels, which led to overlapping electrochemical double layers (EDLs). In 2020 [136], wearable perspiration analyzing sites were used to actively monitor physiological status during exercises without any batteries (Figure 9i). The device is mainly composed of ZnO nanowire (NW) arrays and a flexible PDMS substrate. The working mechanism is based on the coupling of the hydrovoltaic effect and enzymatic reactions. The sweat flowing on ZnO NWs (with lactate oxidase modification) can output a DC electrical signal, and the outputting voltage is dependent on the lactate concentration in sweat as the biosensing signal. Evaporative hydro generators have great potential in alleviating the water–energy crisis. Chen et al. achieved ultra-high voltages of over 100 V and significant freshwater collection based on thermal diffusion-enhanced hydro generators [78]. The integrated system of freshwater and electricity cogeneration, comprising 192 units, exhibits a fresh water collection rate of up to 2.0 L m^−2^ h^−1^ under sunny conditions, as illustrated in Figure 9j.

## 8. The Challenges of Different Solid–Liquid Nanogenerators

**Figure 10:**
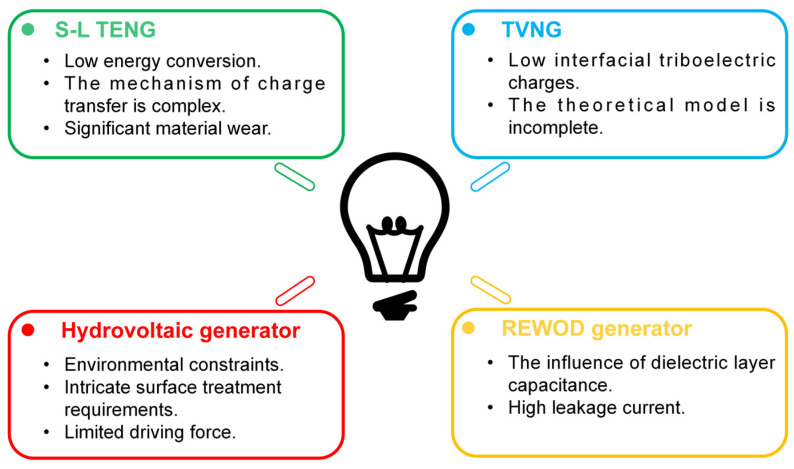
The challenges of the different solid–liquid nanogenerators.

Solid–liquid interaction mechanisms, performance optimization methods, and applications are mentioned in this review. We focused our discussion on different types of solid–liquid nanogenerators, including triboelectrification, hydrovoltaic, reverse electrowetting, and tribovoltaic effects. However, despite significant breakthroughs through research on solid–liquid nanogenerators, some obstacles prevent their widespread application, as depicted in Figure 10 and summarized in this section.

The three bottlenecks of the S-L TENG include the inherently low energy conversion efficiency, the complex mechanism of charge transfer, and the significant material wear at the friction interface.

(1) **Low energy conversion:** In the process of converting mechanical energy into electrical energy, the S-L TENG has the problem of low energy conversion efficiency. Its output power is relatively limited, and it is difficult to meet some application scenarios with high energy demand, which limits its wide application in large-scale energy collection. This is because, in the process of solid–liquid contact electrification and electrostatic induction, there are various energy loss pathways, such as friction loss, charge leakage, etc., resulting in the inability to efficiently convert the input energy into available power output, thus affecting its overall energy conversion efficiency.

(2) **The mechanism of charge transfer is complex:** The charge transfer process at the solid–liquid interface involves the intertwining of various physical and chemical phenomena, and its mechanism is extremely complex. At present, the understanding of how to accurately transfer, distribute, and influence factors of charge at the solid–liquid interface is not deep enough. This leads to the lack of clear theoretical guidance in optimizing the design and performance improvement in the S-L TENG, and it is difficult to improve the structure and materials to improve the charge transfer efficiency, which limits the further improvement in its energy conversion performance.

(3) **Significant material wear:** At the friction interface, the material wear phenomenon is more prominent. Especially under long-term operation or high-frequency working conditions, the loss of materials will gradually increase. This will not only change the characteristics of the friction interface and affect the generation and transmission stability of the charge, but it will also shorten the service life of the equipment and increase the maintenance cost and replacement frequency. Debris generated by wear may also interfere with the normal operation of the equipment, further reducing its performance and reliability.

The two major challenges faced by the TVNG include the low interfacial triboelectric charges and the incomplete theoretical model.

(1) **The low interfacial triboelectric charges:** During the solid–liquid interaction of the TVNG, the frictional charge density generated at the interface is relatively low. This directly leads to the limited amount of charge that can be used for power conversion, thus limiting the size of the output power. The lower interfacial friction charge may be due to the influence of solid–liquid material selection, surface properties, and contact mode, which makes the generation and accumulation of charge insufficient during the friction process and cannot provide sufficient charge basis for efficient energy conversion, thus affecting the power generation performance of the TVNG.

(2) **The theoretical model is incomplete:** The existing theoretical models still have some limitations in explaining the working principle and performance of the TVNG. It is impossible to accurately predict and describe the behavior of the TVNG under different working conditions, such as the output characteristics of different liquid media, solid material combinations, and external conditions. The imperfect theoretical model leads to the lack of a reliable theoretical basis in the development and optimization of the TVNG. It can only be explored through a large number of experiments, which increases the cost and time of research and development and also hinders the rapid development of its technology and the effective improvement in performance.

The challenges faced by the hydrovoltaic generator include environmental constraints, intricate surface treatment requirements, and limited driving force.

(1) **Environmental constraints:** The performance and long-term stability of the hydrovoltaic generator are severely constrained by geographical environmental factors. Different geographical locations have different climatic conditions and water quality. In areas with poor water quality, impurities in the water will adhere to the surface of the equipment, affecting the energy conversion process between the water and equipment. In areas with dry or cold climates, the evaporation rate of water molecules or the mobility of water will be affected, further reducing the driving force. These changes in environmental factors make it difficult for hydrovoltaic generators to achieve efficient and stable power generation in various environments, which limits their applicability and promotion in different regions.

(2) **Intricate surface treatment requirements:** In order to improve the power generation performance of the hydrovoltaic generator, it is often necessary to carry out complex surface treatment, such as designing special nanostructures to enhance the interaction between water and solid surfaces or surface functionalization to regulate the charge transfer process. However, these surface treatment processes usually require high-precision technology and complex operating procedures. This not only requires advanced nano-manufacturing technology to construct suitable surface structures but also requires precise control of surface chemical properties, which increases manufacturing costs and production difficulties. The requirements for production environment and equipment are also high, which limits its large-scale production and wide application.

(3) **Limited driving force:** The driving force provided by the movement or state change of water molecules in the environment is relatively small. For example, in a hydrovoltaic generator driven by water evaporation or natural water flow, the energy that can be converted into electrical energy is limited when the evaporation rate of water molecules is slow or the water flow rate is low. The small driving force makes it difficult for the device to generate a sufficiently high power output, and the energy resources in the surrounding environment cannot be fully utilized, which limits its application potential in large-scale energy supply, and its contribution to the actual energy utilization system is relatively small.

The REWOD generator faces bottlenecks including the influence of dielectric layer capacitance and high leakage current.

(1) **The influence of dielectric layer capacitance:** Improper dielectric layer capacitance will cause efficiency loss in energy storage and release, affect the electric field distribution and charge storage and release capacity, and ultimately reduce energy conversion efficiency and overall performance of the device.

(2) **High leakage current:** High leakage current will cause a lot of power loss inside the equipment, which reduces the power output that can be effectively utilized. This part of the leakage current not only wastes energy but also may cause local heating of the equipment, affecting the stability and safety of the equipment. At the same time, the high leakage current also makes it difficult for the equipment to meet some application scenarios with high requirements for power quality, which limits its application in the power supply of precision electronic equipment.

## 9. Conclusion and Perspectives

Looking forward to the future, the development of solid–liquid nanogenerator technology will mainly focus on the following aspects:

(1) The circuit management and structural design should be enhanced to optimize the output performance of the solid–liquid nanogenerators system.

(2) Reaching a larger scale of energy harvesting and utilization is made possible by the integration of solid–liquid nanogenerators through meticulous design and technical strategies.

(3) A comprehensive investigation into the multi-field coupling mechanism in solid–liquid nanogenerators not only facilitates the enhancement in energy conversion efficiency but also offers novel insights for the advancement of intelligent materials and systems.

In recent years, significant progress has been made in energy harvesting technology based on solid–liquid nanogenerators, showing the growing importance in the energy field. In this paper, the application of various solid–liquid nanogenerators in energy collection is described in detail, and their respective advantages and disadvantages are shown in Table 2. The S-L TENG has the advantages of wide material selection, strong environmental adaptability, a wide operating frequency range, and a relatively simple structure. However, it faces problems such as low charge density limiting conversion efficiency, friction and wear and heat affecting equipment life, and complex charge transfer. The TVNG does not require additional circuits and can operate under low mechanical forces, but it has the disadvantages of low conversion efficiency, difficulty in large-scale applications, and less interface charge affecting power generation. The hydrovoltaic generator can work continuously in the water environment. It is simple in design and light in structure, but it has low output power, large environmental constraints, and complex surface treatment. The REWOD generator combines rotation and water flow to increase energy output, which is suitable for high-pressure applications, but the mechanical components are complex, easy to wear, and have poor long-term reliability.

## Figures and Tables

**Table 1:** The nanomaterial engineering of solid–liquid nanogenerators in recent years.

Type	Nanomaterials	Year	Power Density	References
S-L TENG	Nanoparticles (AgNPs)	2020	7.7V	[95]
	Nanofiber (TPU)	2024	282V	[98]
	Nanowire array (PS)	2019	18.4V	[99]
Hydrovoltaic generator	Nanoparticles (Al_2_O_3_/CB)	2022	5.86V	[100]
	Nanofiber (NNF)	2024	4.82V	[101]
	Nanowire array (SiNWs)	2024	1.28V	[102]

**Table 2:** Comparative analysis of the advantages and disadvantages associated with various types of solid–liquid nanogenerators.

Type	Strengths	Weaknesses
S-L TENG	• Wide selection of materials. • Environment adaptability. • Wide operating frequency range. • The structure is relatively simple.	• The low charge density restricts the conversion efficiency. • The wear and heat are heavy, which affects the service life of the equipment. • The charge transfer is complex.
TVNG	• No additional circuitry required. • Can be operated at low mechanical forces.	• Low conversion efficiency. • It is difficult to achieve large-scale applications. • The interface charge is less, affecting power generation.
Hydrovoltaic generator	• Can work continuously in the water environment. • The design is relatively simple. • Lightweight structure.	• Low output power. • Constrained by the environment; the scope of application is limited. • Complex surface treatment.
REWOD generator	• Combined with rotation and water flow, energy output improves. • Suitable for high-voltage applications.	• Complex mechanical components. • The device is easy to wear. • Poor long-term reliability.
